# Antinociceptive and Anti-Inflammatory Activity from Algae of the Genus *Caulerpa*

**DOI:** 10.3390/md9030307

**Published:** 2011-03-02

**Authors:** Carolina Babosa Brito da Matta, Éverton Tenório de Souza, Aline Cavalcanti de Queiroz, Daysianne Pereira de Lira, Morgana Vital de Araújo, Luiz Henrique Agra Cavalcante-Silva, George Emmanuel C. de Miranda, João Xavier de Araújo-Júnior, José Maria Barbosa-Filho, Bárbara Viviana de Oliveira Santos, Magna Suzana Alexandre-Moreira

**Affiliations:** 1 LaFI—Laboratory of Pharmacology and Immunity, Institute of Biological Sciences and Health, Federal University of Alagoas, 57020-720, Maceió, AL, Brazil; E-Mails: caroll_brito@hotmail.com (C.B.B.M.); evertontenorio_al@yahoo.com.br (E.T.S.); allycq_farmacia@hotmail.com (A.C.Q.); morgana_vital@hotmail.com (M.V.A.); luiz0710@gmail.com (L.H.A.C.-S.); 2 Laboratory of Technology Pharmaceutical, Federal University of Paraíba, 58051-900, João Pessoa, PB, Brazil; E-Mails: daysianneplira@yahoo.com.br (D.P.L.); jbarbosa@ltf.ufpb.br (J.M.B.-F.); 3 Laboratory of Marine Algae, Department of Systematics and Ecology, Federal University of Paraíba, 58051-900, João Pessoa, PB, Brazil; E-Mail: mirandag@dse.ufpb.br; 4 Laboratory of Research in Natural Resources, Institute of Chemistry and Biotechnology, Federal University of Alagoas, 57072-970, Maceió, AL, Brazil; E-Mail: joaoxjr@yahoo.com.br

**Keywords:** antinociceptive, anti-inflammatory, *Caulerpa mexicana*, *Caulerpa sertularioide*, marine algae

## Abstract

Marine natural products have been the focus of discovery for new products of chemical and pharmacological interest. The aim of this study was to evaluate the antinociceptive activity of the methanolic (ME), acetate (AE), hexanic (HE) and chloroform (CE) extracts obtained from *Caulerpa mexicana*, and ME, CE and HE obtained from *Caulerpa sertularioides*. These marine algae are found all over the world, mainly in tropical regions. Models such as the writhing test, the hot plate test and formalin-induced nociception test were used to evaluate antinociceptive activity in laboratory mice. In the writhing test, all the extracts were administered orally at a concentration of 100 mg/kg, and induced high peripheral antinociceptive activity, with a reduction in the nociception induced by acetic acid above 65%. In the hot plate test, treatment with extracts from *C. sertularioides* (100 mg/kg, p.o.) did not significantly increase the latency of response, although the ME, AE and HE from *C. mexicana* showed activity in this model. This result suggests that these extracts exhibit antinociceptive activity. In the formalin test, it was observed that ME, AE and HE obtained from *C. mexicana* reduced the effects of formalin in both phases. On the other hand only CE from *C. sertularioides* induced significant inhibition of the nociceptive response in the first phase. To better assess the potential anti-inflammatory activity of the extracts, the carrageenan-induced peritonitis test was used to test *Caulerpa* spp. extracts on cell migration into the peritoneal cavity. In this assay, all extracts evaluated were able to significantly inhibit leukocyte migration into the peritoneal cavity in comparison with carrageenan. These data demonstrate that extracts from *Caulerpa* species elicit pronounced antinociceptive and anti-inflamatory activity against several nociception models. However, pharmacological and chemical studies are continuing in order to characterize the mechanism(s) responsible for the antinociceptive action and also to identify the active principles present in the *Caulerpa* species.

## 1. Introduction

The search for pharmacological properties from natural products has led to the discovery of pharmacologically active substances, with important applications both in the experimental field and identification of active principles with therapeutic interest [1–5]. Currently, about 25–30% of all active principles used in treatments are extracted from natural products [6]. The plant kingdom is responsible for the largest share of chemical diversity recorded in the literature to date and has contributed quite significantly to the research and discovery of new drugs of natural origin, as well as the supply of substances useful for treating diseases that affect living beings [7–11]. However, it should be noted that marine natural products have also been the focus of discovery of new products of chemical and pharmacological interest [12–18].

Nonetheless, only about 20% of natural products from around the world have had their extracts submitted to pharmacological or biological tests [19]. To make matters worse, extinction of many species has become more frequent, with an estimated thousand species becoming extinct each year on the planet. Many of these species have not yet even been described, cataloged or studied [20].

Marine organisms are sources of numerous new compounds with multiple pharmacological properties [21]. The variety and complexity of small molecules which are the secondary metabolites of plants and marine organisms is difficult to be obtained by chemical synthesis by laboratory methods, these being the direct result of millions of years of evolution, reaching highly refined forms for protection against the weather and resistance to climate, pollution and predators [22].

In general, algae synthesize secondary metabolites such as terpenoids [23], alkaloids [24], flavonoids [25], tannins [26] and acetogenins [27]. Polar polyphenols may also occur in high concentrations [28,29]. In the literature, different pharmacological activities of macroalgae have been reported, including: antibacterial [30,31], antitumoral [32,33], anti-angiogenic [34,35], antiviral [36,37], antileishmania [38] and antioxidant [39] activity. However, in Brazil, this research field has not been well explored, despite the wealth of our marine flora.

Recently, the hypothesis that *Caulerpa* species (Chlorophyta, order Caulerpales, family Caulerpaceae) produce secondary metabolites with possible antinociceptive actions was investigated. In our preliminary investigation of the crude methanolic extract and phases from another specie from algae, *Caulerpa racemosa*, our group showed that this specie also had antinociceptive activity in the same models described in this work [40]. Although marine algae are an important source for biologically active natural products, few studies, specifically in Brazil, have been conducted with the the purpose of evaluating antinociceptive and antiinflamatory activity in animal models. As such, this study intended to evaluate the antinociceptive activity of extracts of macroscopic green algae *Caulerpa mexican* and *Caulerpa sertularioides* in murine models.

## 2. Results and Discussion

The antinociceptive potential of extracts from algae of the genus *Caulerpa* was evaluated using three well-accepted murine pain models, namely acetic acid-induced writhing, hot plate and formalin-induced nociception tests. The acetic acid-induced abdominal writhing and hot plate test have been reported to be useful to investigate peripheral and central activity, respectively, while the formalin-induced nociception test is valuable in detecting both effects.

Pre-treatment for all *Caulerpa* species resulted in significant inhibition of the acetic acid-induced writhing response. All extracts were evaluated at a dose of 100 mg/kg. The methanolic (ME), acetate (AE), hexanic (HE) and chloroform (CE) extracts from *C. mexicana* induced high peripheral antinociceptive activity with an inhibition of 78.4%, 73.2%, 83.1% and 77.0%, respectively. The pharmacological evaluation of extracts of *C. sertularioides* in the writhing test showed that the HE, CE, AE and ME induced inhibition of 66.5%, 67.0%, 60.7% and 67.0%, respectively. Moreover, these protective effects in the writhing test were also observed for dypirone (86.5%), as expected, used as the reference peripheral analgesic drug (Table 1). These data suggest that there is a possible antinociceptive action for extracts of *C. Mexican* and *C. sertularioides.* These data also corroborate a previous study on antinociceptive activity of *Caulerpa racemosa* [40].

The writhing test is commonly used for screening peripherally active analgesic. Algogenic agents, such as acetic acid, provoke a stereotypical behavior in mice characterized by abdominal contractions, movements of the body as a whole, twisting of dorso abdominal muscles and a reduction in motor activity and in motor coordination [41]. This model involves different nociceptive mechanisms, such as release of biogenic amines (e.g., histamine and serotonin), cyclooxygenases and their metabolites (e.g., PGE_2_ and PGF2α) [42] and opioid mechanisms [43]. Furthermore, it is well established that the nociceptive response caused by acetic acid is also dependent on the release of some cytokines, such as TNF-α, interleukin 1β and interleukin 8 via modulation of macrophages and mast cells localized in the peritoneal cavity [44]. In spite of compounds that act peripherally presenting action in this model, such as local anesthetics, muscle relaxants, ansiolitic, tranquilizers, among others, this remains being a good model to investigate the central analgesic action of substances. Because of this, the hot plate test was carried out with the aim of evaluating whether the *Caulerpa* species could demonstrate an antinociceptive effect.

In the hot plate test, the extracts of *C. sertularioides* did not significantly increase the latency of response, indicating that they do not show central activity (data not shown). On the order hand, treatment with ME, AE, CE and HE from *C. mexicana* (Table 2) caused a marked increase in the latency time of the animals at the times of 90 and 150 (5.4 ± 0.6 s and 5.4 ± 0.4 s), 90 and 150 (5.3 ± 0.9 s and 5.9 ± 1.3 s), 90 (5.7 ± 0.7 s) and 90–120 (5.8 ± 0.9 s; 5.9 ± 1.00 s) minutes respectively. These results suggest the action of analgesic activity for *C. mexicana* may be mediated through inhibition of pain receptors or inhibition of mediators like ciclooxigenase (*i.e.*, COX-3). The treatment with morphine (4.3 mg/kg, s.c.), the opioid receptor agonist, induced a significant increase in latency time in the hot plate test, as expected, which persisted for at least 150 min. The hot plate test is considered to be selective for centrally acting analgesic compounds, like morphine, while peripheral analgesics are known to be inactive on this kind of painful stimulus [45]. Although an effect in the hot plate test was observed, we cannot say that the effect of this extract is mediated centrally.

**Figure 1.**
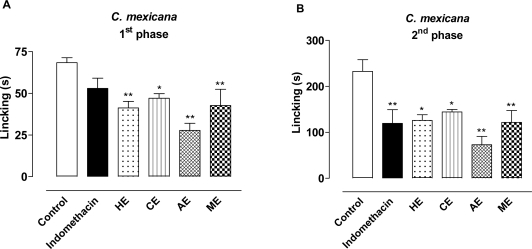

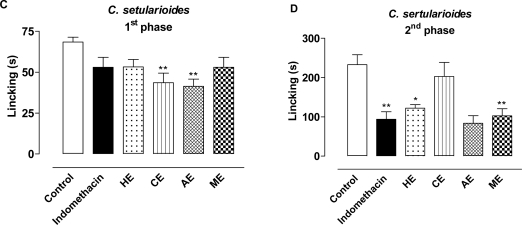
Effect of extracts of algae *C. mexicana*, *C. sertularioides* and indomethacin administered orally at a dose of 100 mg/kg in the formalin test (6 animals). (**A**) and (**C**) represents 1st phase, (**B**) and (**D**) represents 2nd phase. Statistical differences between the treated and the control groups were evaluated using ANOVA and Dunnett tests and the asterisks denote the significance levels in comparison with control groups, * *P* < 0.05, ** *P* < 0.01.

Neurogenic and inflammatory pain were evaluated using the formalin test. The first phase corresponds to acute neurogenic pain, while the second phase corresponds to inflammatory pain. The first and second phases are generally believed to reflect excitation of peripheral afferent nociceptors and central sensitization, respectively [46,47]. Substance P and bradykinin participate in the first phase, while serotonin, histamine, bradykinin, nitric oxide and prostaglandins are involved in the second phase [48]. Different mechanisms have been shown to be involved in first and second phase nociceptive behaviors, based on the differential pharmacology associated with these behaviors. For example, while second phase behaviors are selectively attenuated by cyclooxygenase inhibitors, first and second phase behaviors are attenuated by opioids [47]. Treatment with HE, CE, AE and ME from *C. mexicana* induced an inhibition of 39.7%, 31.1%, 60.2% and 50.2%, respectively, in the first phase (Figure 1A). Furthemore, all extracts of *C. mexicana* induced significant inhibition in the second phase, with an inhibition of 47.7% (ME), 68.7% (AE) 45.8% (HE) and 37.9% (CE) (Figure 1B). The pharmacological evaluation of extracts of *C. sertularioides* in the formalin test showed that the CE and AE could reduce the duration (36.2% and 40.0% inhibition) of paw licking in the first phase (Figure 1C). On the other hand, in the second phase CE was not active, while ME, AE and HE, were able to decrease the time that animals spent licking the injected paw (55.8%, 64% and 47.5% inhibition, respectively) (Figure 1D). Treatment with indomethacin, the NSAID significantly inhibited formalin induced nociception in the second phase (48.7% inhibition), but not the first phase. Thus, the results shown in the first phase of the model corroborate with those obtained in the writhing test, confirming the antinociceptive activity of the extracts.

**Figure 2.**
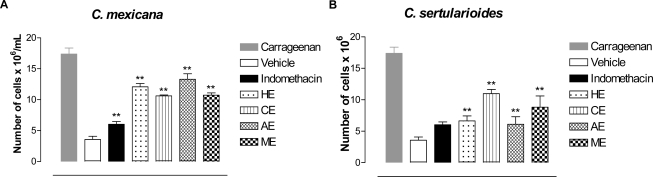
The effect of *Caulerpa* spp. extracts on cell migration. *Caulerpa* spp. extracts (100 mg/kg, p.o.) and indomethacin (35.7 mg/kg, p.o.) were evaluated using the carrageenan-induced peritoneal inflammation test. Each point represents the mean ± S.E.M. of six animals. Statistical differences between the treated and the control groups were evaluated by ANOVA and Dunnett tests, and the asterisks denote the significance levels in comparison with control groups, * *P* < 0.05, ** *P* < 0.01.

Since reduction of the second phase in the formalin test implied a possible anti-inflammatory mechanism, and considering previous studies from our group [40] that have demonstrated that another specie of *Caulerpa* has anti-inflammatory activity, we decided to evaluate the activity of these species in models of cell migration. To determine the effects of extracts from *Caulerpa* spp. on peritoneal inflammation induced by carrageenan, the mice were treated with test samples. Using carrageenan as a stimulus, it was possible to produce an acute inflammatory response after 4 h in the peritoneal cavity of mice, with a large number of leukocytes in the exudates. With the aim of evaluating a possible inhibitory effect of *Caulerpa* spp. extracts on cell migration into the peritoneal cavity, the carrageenan-induced peritonitis test was used. In this assay, all extracts evaluated were able to significantly inhibit leukocyte migration into the peritoneal cavity in comparison with carrageenan. The HE, CE, AE and ME from *C. mexicana* inhibited 30.5%, 38.9%, 23.5%, 38.26%, respectively (Figure 2A). While the extracts from *C. sertularioides* induced inhibition of 61.8% (HE), 36.9%, (CE), 71.7% (AE) and 49.25% (ME) (Figure 2B). The treatment with indomethacin inhibited 65.4% leukocyte migration.

## 3. Experimental Section

### 3.1. Extraction and Isolation

The algae *C. mexicana* and *C. sertularioide* were collected from the coastal region of Bessa (7°03′52″S/34°49′51″W), João Pessoa, Paraíba State, Brazil in April 2008. The specimens were identified by Dr. George Emmanuel Cavalcanti de Miranda. Voucher specimens of *C. mexicana* (JPB 13985), *C. sertularioides* (JPB 13983) have been deposited in the Lauro Pires Xavier Herbarium at the Federal University of Paraíba (Universidade Federal da Paraíba), Brazil. The fresh algae were lyophilized and exhaustively extracted with hexane, chloroform, ethyl acetate, methanol and water in a Soxhlet apparatus, to obtain the respective extracts.

### 3.2. Biological Activity Tests

#### 3.2.1. Drugs and Reagents

The following drugs and reagents were used: acetic acid (Merck), dipyrone (Sigma Chemical), morphine sulfate (Dimorf-Cristalia-BR), indomethacin (Merck), arabic gum (Sigma Chemical) and Tween 20 (Sigma). A solution of formalin 2.5% was prepared with formaldehyde (Merck) in saline (NaCl 0.9%). The botanical material was used as suspensions in Tween 80 (s.q.f.) and arabic gum (vehicle) in all the experiments and were administered by oral route at a dose of 100 mg/kg. Dipyrone, morphine and indomethacin were used as reference drugs. Dipyrone and indomethacin were administered by oral route and morphine by subcutaneous route. The control group was composed of the vehicle (arabic gum).

#### 3.2.2. Animals

All experiments were performed with male and female Swiss mice (20–25 g). Animals were maintained in a room at a controlled temperature of 22 ± 2 °C for 12-h light/dark cycle with free access to food and water. Eight hours before each experiment animals received only water, in order to avoid food interference with substance absorption. Animal care and research protocols were in accordance with the principles and guidelines for the care of laboratory animals and the ethical guidelines for investigations of experimental pain in conscious animals [49]. The experiments were performed with the approval of the protocol by the local Institutional Ethics Committee-UFAL (No. 006443/2005-78).

#### 3.2.3. Acetic Acid-Induced Writhing Test

The writhing test was carried out as described by Koster *et al.* [50]. Groups of mice (*n* = 6) were treated with the methanolic (ME), ethyl acetate (AE), hexanic (HE) and chloroform (CE) extracts from *Caulerpa* species (100 mg/kg, p.o.), dipyrone (40 mg/kg, p.o.) and the vehicle (p.o.). The writhings were induced by intraperitoneal injection with a 0.6% acetic acid solution (0.1 mL/10 g) 40 min after treatment. The number of writhings were counted starting at 5 min after injection of the stimulus for 20 min. Antinociceptive activity was expressed as percent inhibition of the usual number of writhings observed in control animals.

#### 3.2.4. Hot Plate Test

The hot plate test was performed following the method of Eddy and Leimbach [51]. Different groups of animals (*n* = 6) received ME, AE, HE and CE from *Caulerpa* species (100 mg/kg, p.o.), morphine (4.3 mg/kg, s.c.) and the vehicle (0.5 mL, p.o.) Then, mice were placed on the equipment, which was maintained at 55 ± 1 °C, and the reaction time was noted by observing either the licking of the fore and hind paws or jumping at 30, 60, 90, 120 and 150 min after administration of the extracts. The baseline was considered as the mean reaction time obtained at 30 min and 60 min before administration of the phases, compounds or morphine and was defined as the normal reaction of the animal to temperature. The cut-off time used to prevent skin damage was 15 s.

#### 3.2.5. Formalin-Induced Nociception

The formalin test was carried out as described by Hunskaar, Hole and Tjølsen *et al.* [52,53]. Animals received a dose of 20 μL of a 2.5% formalin solution (0.92% formaldehyde, in saline) on the ventral surface of the right hind paw. Animals were observed from 0 to 5 min (neurogenic phase) and from 15 to 30 min (inflammatory phase) and the time that they spent licking the injected paw was recorded and considered as indicative of nociception. Animals received ME, AE, HE and CE from *Caulerpa* species (100 mg/kg, p.o.) or the standard drug (indomethacin, 35.7 mg/kg, p.o.) 40 min before formalin injection. Control animals received only the vehicle (arabic gum).

#### 3.2.6. Carrageenan-Induced Peritonitis in Mice

For this series of experiments, the method described by Ferrándiz and Alcaraz [54] was used. Carrageenan (Sigma Aldrich) was freshly prepared (10 mg/mL) in sterile 0.9% w/v saline, and 250 μL was injected i.p. After 4 h, the animals were killed by cervical dislocation. The peritoneal cavity was washed with 1.5 mL cold PBS, and after gentle manual massage, the exudate was retrieved and its volume measured. The number of recruit leukocytes to the peritoneum was counted in a Neubauer chamber and results were expressed as cells × 10^6^/mL. The exudate was collected and used freshly for cell counts and cytospin preparations. The *Caulerpa* extracts (100 mg/kg, p.o.), the carrageenan group (arabic gum, p.o.) and the reference drug (indomethacin, 35.7 mg/kg, p.o.) were administered 30 min before the carrageenan injection. In the negative control group, animals just received the same dose of a vehicle (arabic gum, p.o.) 30 min before the saline injection by intraperitoneal route.

### 3.3. Statistical Analysis

Data are reported as mean ± S.E.M. and were analyzed statistically using analysis of variance (ANOVA) followed by Dunnett’s test. Results with *P* < 0.05 were considered significant (* *P* < 0.05, ** *P* < 0.01).

## 4. Conclusions

In conclusion, this study has shown that extracts from *Caulerpa* species have significant antinociceptive and anti-inflammatory effects in laboratory animals at the dose and route investigated. It can be argued that the extracts have antinociceptive activity and may possibly act via inhibition of inflammatory mediators. However, pharmacological and chemical studies are needed in order to characterize the mechanism(s) responsible for the antinociceptive and anti-inflammatory action and also to identify other active agents present in this plant. Moreover, the results obtained in this work contribute significantly to the pharmacological studies from marine products.

## Figures and Tables

**Table 1.** The antinociceptive effects of extracts from algae *C. mexicana* (100 mg/kg, p.o.), *C. sertularioides* (100 mg/kg, p.o.) and dipyrone (40 mg/kg, i.p.) in the acetic acid-induced writhing model in mice.

	***C. mexicana***		***C. sertularioides***	

	**Number of writhing**			

**Treatment**	**Mean ± S.E.M. ^a^**	**I (%) ^b^**	**Mean ± S.E.M. ^a^**	**I (%) ^b^**
**Vehicle**	35.5 ± 1.6	–	34.9 ± 1.9	–
**Dypirone**	8.0 ± 2.3	86.5 **	5.9 ± 1.7	83.2 **
**HE**	6.0 ± 1.4	83.1 **	11.7 ± 2.0	66.5 **
**CE**	8.2 ± 2.3	77.0 **	11.5 ± 0.9	67.0 **
**AE**	9.5 ± 1.4	73.2 **	13.7 ± 2.8	60.7 **
**ME**	7.7 ± 0.7	78.4 **	11.5 ± 1.8	67.0 **

a Represents the Mean ± S.E.M. of 6 animals. Statistical differences between the treated and the control groups were evaluated by ANOVA and Dunnett tests and the asterisks denote the significance levels in comparison with control groups;

** *P* < 0.01;

b Represents percentage inhibition. HE, hexanic; CE, chloroform; AE, acetate; ME, methanolic.

**Table 2.** Time-course for response with treatment of *C. mexicana* extracts (100 mg/kg, p.o.) and morphine (4.3 mg/kg, s.c.) on thermal nociception (hot plate test).

	**Post-treatment (min) ^a^**					
**Animal Group**	**0 min**	**30 min**	**60 min**	**90 min**	**120 min**	**150 min**
	**Time latency (s)**					
**Control**	1.4 ± 0.3	2.2 ± 0.6	1.8 ± 0.2	3.2 ± 0.3	2.8 ± 0.3	2.6 ± 0.5
**Morphine**	6.9 ± 0.4	5.8 ± 0.3	12.8 ± 0.4 **	10.3 ± 0.8 **	9.7 ± 0.7 **	9.7 ± 0.9 **
**ME**	3.5 ± 0.5	3.0 ± 0.4	4.5 ± 0.3	5.4 ± 0.6 *	5.1 ± 0.8	5.4 ± 0.4 *
**AE**	1.7 ± 0.4	3.4 ± 0.3	3.1 ± 0.5	5.3 ± 0.9 **	4.1 ± 0.5	5.9 ± 1.3 **
**CE**	2.7 ± 0.2	3.7 ± 0.5	4.4 ± 1.0	5.7 ± 0.7 *	3.6 ± 0.6	3.9 ± 1.0
**HE**	2. 7 ± 0.3	2.8 ± 0.6	4.6 ± 0.7	5.8 ± 0.9 *	5.9 ± 1.0 *	5.1 ± 0.8

a Results represents time latency in second (s). Represents the mean ± S.E.M. of 6 animals;

* *P* < 0.05;

** *P* < 0.01 (ANOVA and Dunnett tests were used to evaluate the significance levels in comparison to time-zero).
